# A Large Palmar Atypical Lipomatous Tumor Extending Into the Carpal Tunnel and Guyon’s Canal: Challenges in Nerve-Sparing Surgical Excision

**DOI:** 10.7759/cureus.115986

**Published:** 2026-09-09

**Authors:** Merna Elsharawy, Mohammad Mohammad

**Affiliations:** 1 Burns and Plastic Surgery, Manchester University NHS Foundation Trust, Manchester, GBR

**Keywords:** atypical lipomatous tumour, carpal tunnel surgery, en bloc excision, guyon's canal, hand sarcoma, mdm2 amplification, microsurgical dissection, sarcoma mdt

## Abstract

We report a complex case of a large atypical lipomatous tumor (ALT) extending into the carpal tunnel and Guyon’s canal, highlighting the diagnostic considerations, anatomical challenges, and principles of safe surgical excision. A 67-year-old female presented with a progressively enlarging swelling involving the thenar, mid-palmar, distal palmar, and wrist regions. Examination revealed a large palmar mass without neurovascular deficit or muscle wasting. MRI demonstrated a 63 × 27 × 79 mm lipomatous lesion arising from the volar wrist and extending into the carpal tunnel and Guyon’s canal, with internal septations and mild heterogeneity, raising suspicion for an ALT versus low-grade liposarcoma. Following the Sarcoma Multidisciplinary Team (MDT) discussion, surgical excision was planned under the plastic surgery department. A volar approach was used. The lesion was predominantly superficial in the radial palm but extended deeply into Guyon’s canal, where it was closely related to the ulnar neurovascular bundle and abutted the median nerve within the carpal tunnel, with the radial neurovascular bundle incorporated within the tumor pseudocapsule. Careful microsurgical dissection was required to separate the tumor from the common digital nerves while preserving both the median and ulnar nerve branches. En bloc excision was achieved with no residual tumour. Histology confirmed an ALT. The patient recovered well and was discharged the same day. This case highlights the complexity of large palmar lipomatous tumors extending into confined neuroanatomical spaces. Preoperative MRI and MDT planning were essential in guiding the operative strategy. The case emphasises meticulous dissection and nerve preservation while achieving complete en bloc oncological excision and maintaining hand function, making it a valuable learning case.

## Introduction

Atypical lipomatous tumor (ALT), also termed well-differentiated liposarcoma (WDLPS), is classified by the World Health Organization (WHO) as an intermediate (locally aggressive) adipocytic neoplasm. Although ALT and WDLPS are histologically synonymous, the term ALT is preferentially used for surgically resectable tumors arising in the extremities and trunk. These tumors have no metastatic potential unless dedifferentiation occurs, but they remain locally aggressive with a risk of local recurrence, necessitating accurate diagnosis and appropriate surgical management [1].

ALTs are uncommon, locally aggressive adipocytic neoplasms that rarely arise in the hand. Benign lipomas involving the carpal tunnel and Guyon’s canal have previously been described, including cases causing simultaneous median and ulnar nerve compression [2,3]. However, the presentation of an atypical lipomatous tumor extending into both the carpal tunnel and Guyon’s canal, with a close anatomical relationship to the median and ulnar neurovascular structures, represents a particularly challenging surgical presentation.

## Case presentation

A 67-year-old right-handed woman presented with an 18-month history of a progressively enlarging left palmar swelling. Clinical examination demonstrated a large volar hand mass involving the thenar, mid-palmar, distal palmar, and wrist regions without neurological deficit, muscle wasting, or restriction of movement. MRI demonstrated a 63 × 27 × 79 mm lipomatous lesion arising from the volar wrist and extending into both the carpal tunnel and Guyon’s canal (Figures 1-2). Internal septations and mild signal heterogeneity raised concern for an ALT or low-grade liposarcoma. Following review by the Regional Sarcoma Multidisciplinary Team (MDT), surgical excision was recommended.

**Figure 1:**
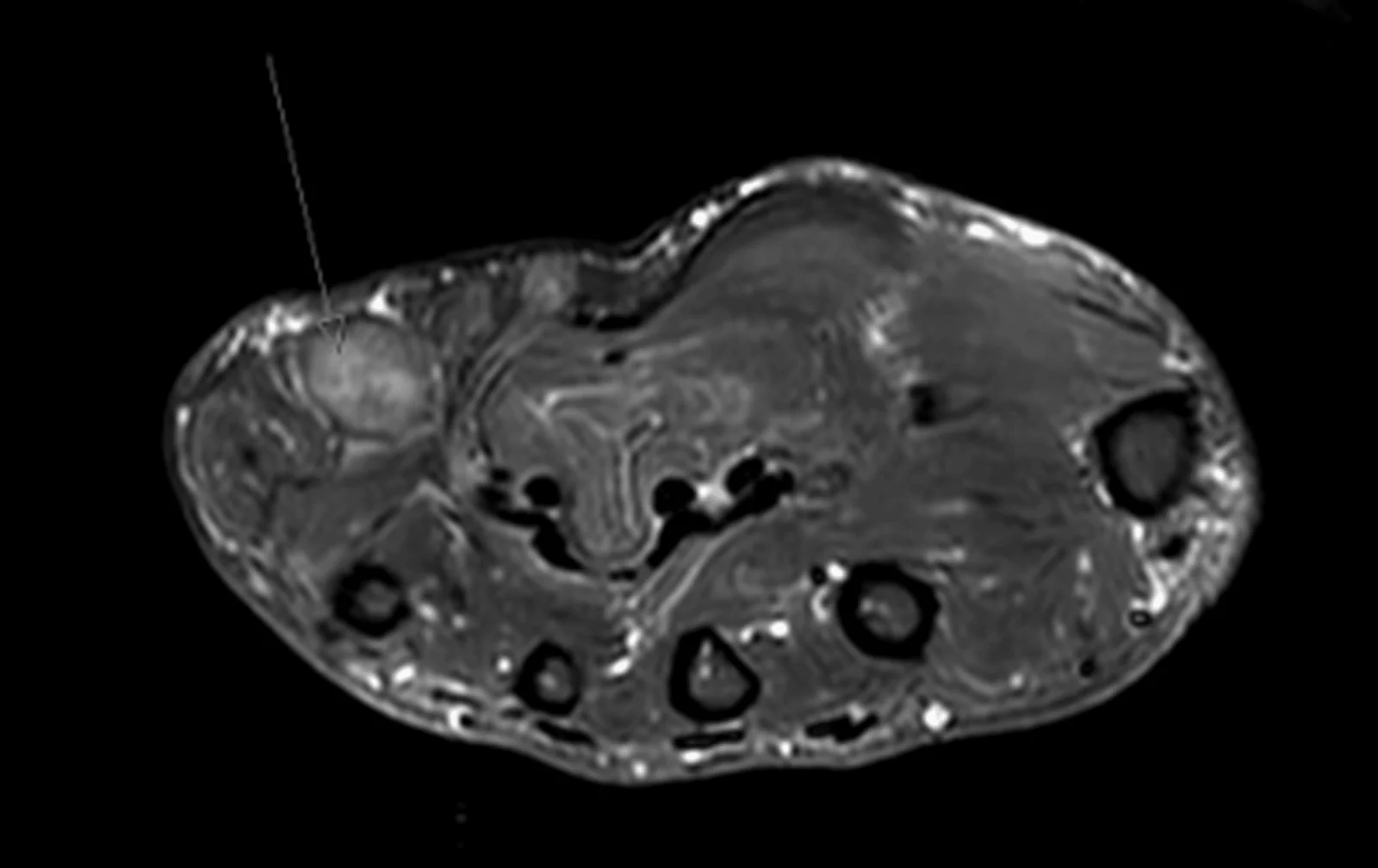
Axial MRI demonstrating a large volar lipomatous tumor (arrow) extending into the carpal tunnel and Guyon's canal.

**Figure 2:**
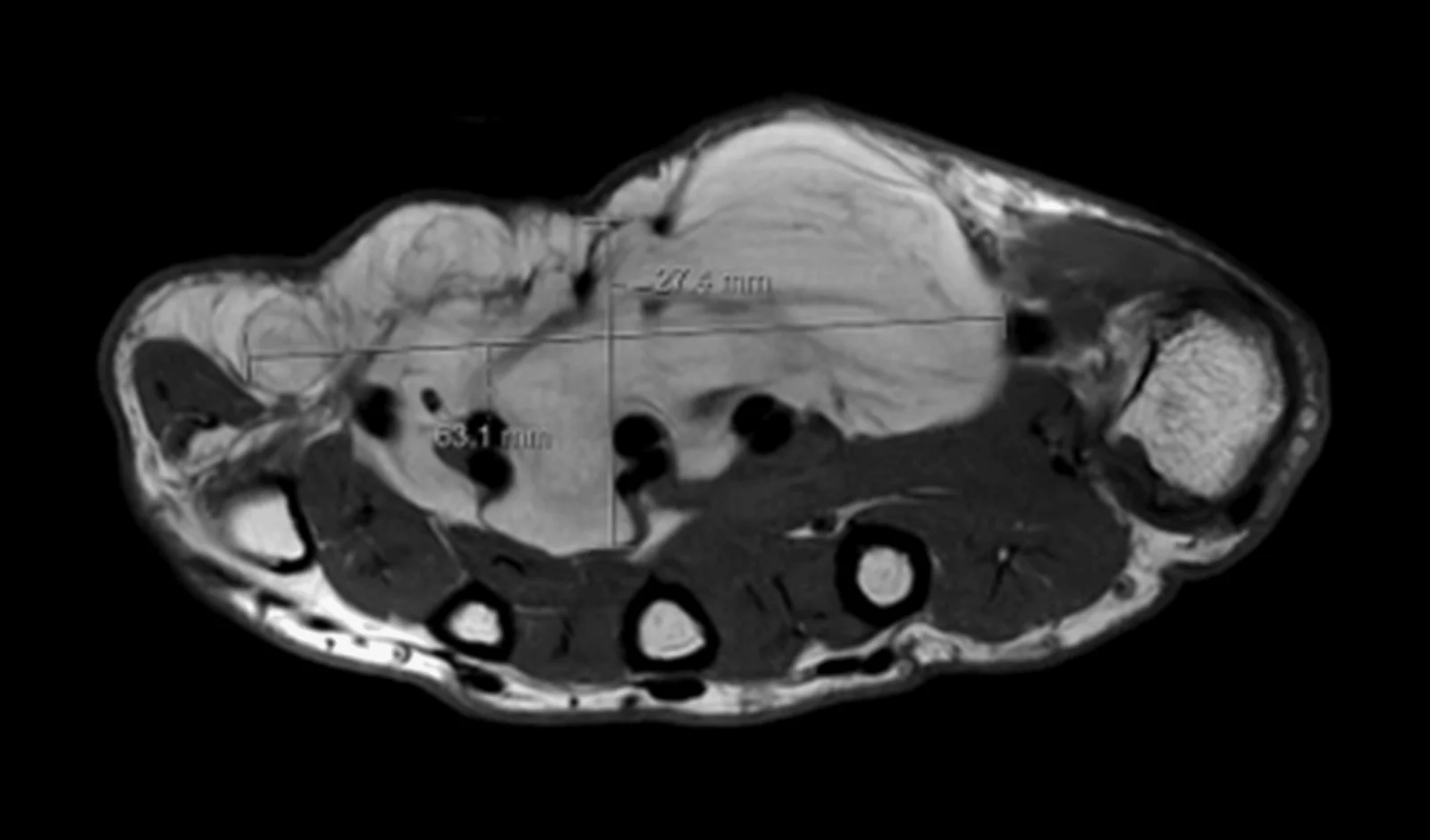
Preoperative axial MRI demonstrating the dimensions and extent of the volar hand atypical lipomatous tumor.

Intervention

Excision was performed through a volar approach. Intraoperatively, the tumor occupied the radial palm and extended deeply into Guyon’s canal, where it was closely associated with the ulnar neurovascular bundle (Figure 3). The radial neurovascular bundle was incorporated within the tumor pseudocapsule and required careful microsurgical dissection for preservation. Meticulous dissection enabled preservation of the common digital nerves and surrounding neurovascular structures (Figure 4). Complete en bloc excision was achieved without residual palpable disease.

**Figure 3:**
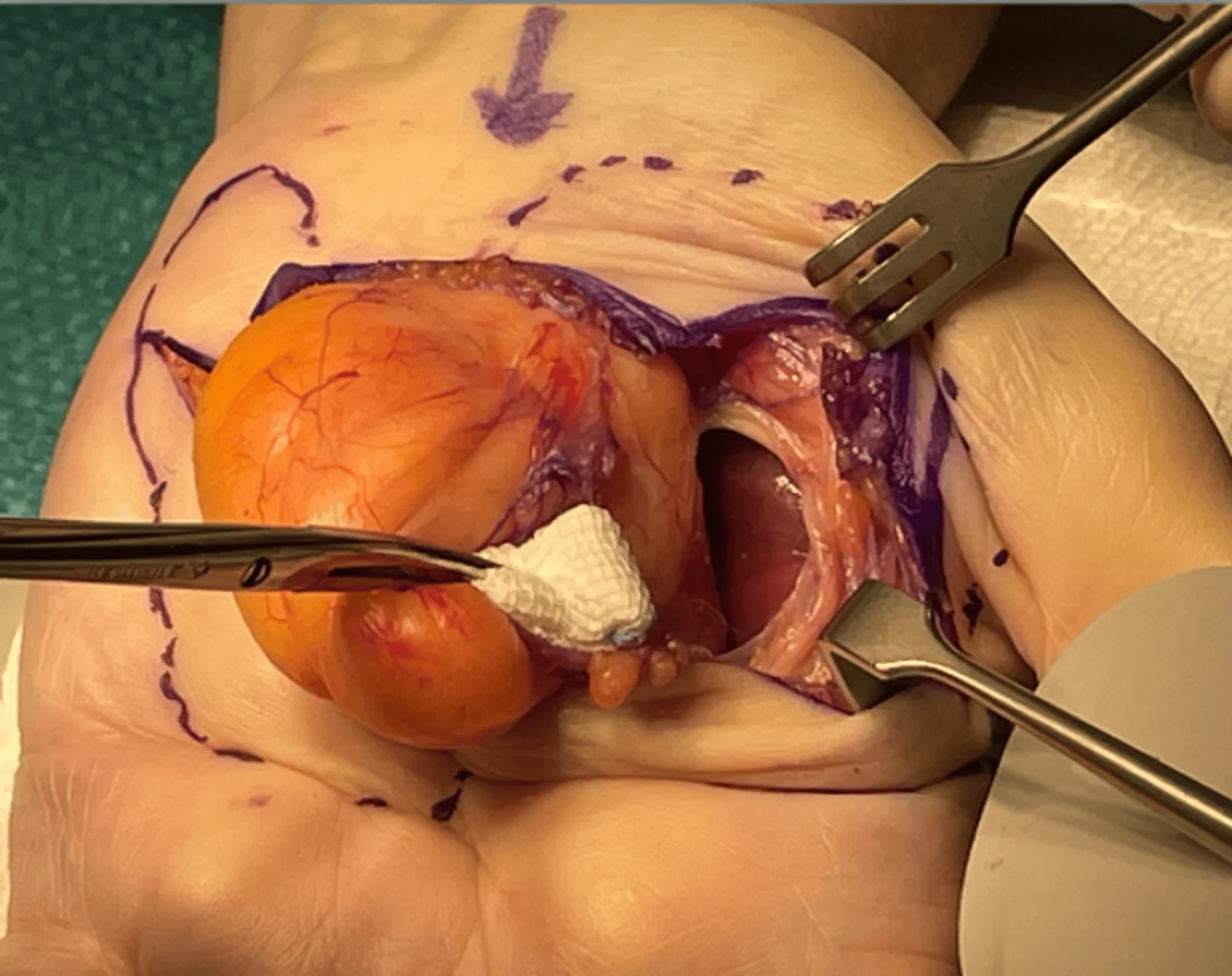
Intraoperative photograph demonstrating the tumor extending into Guyon's canal during nerve-sparing dissection.

**Figure 4:**
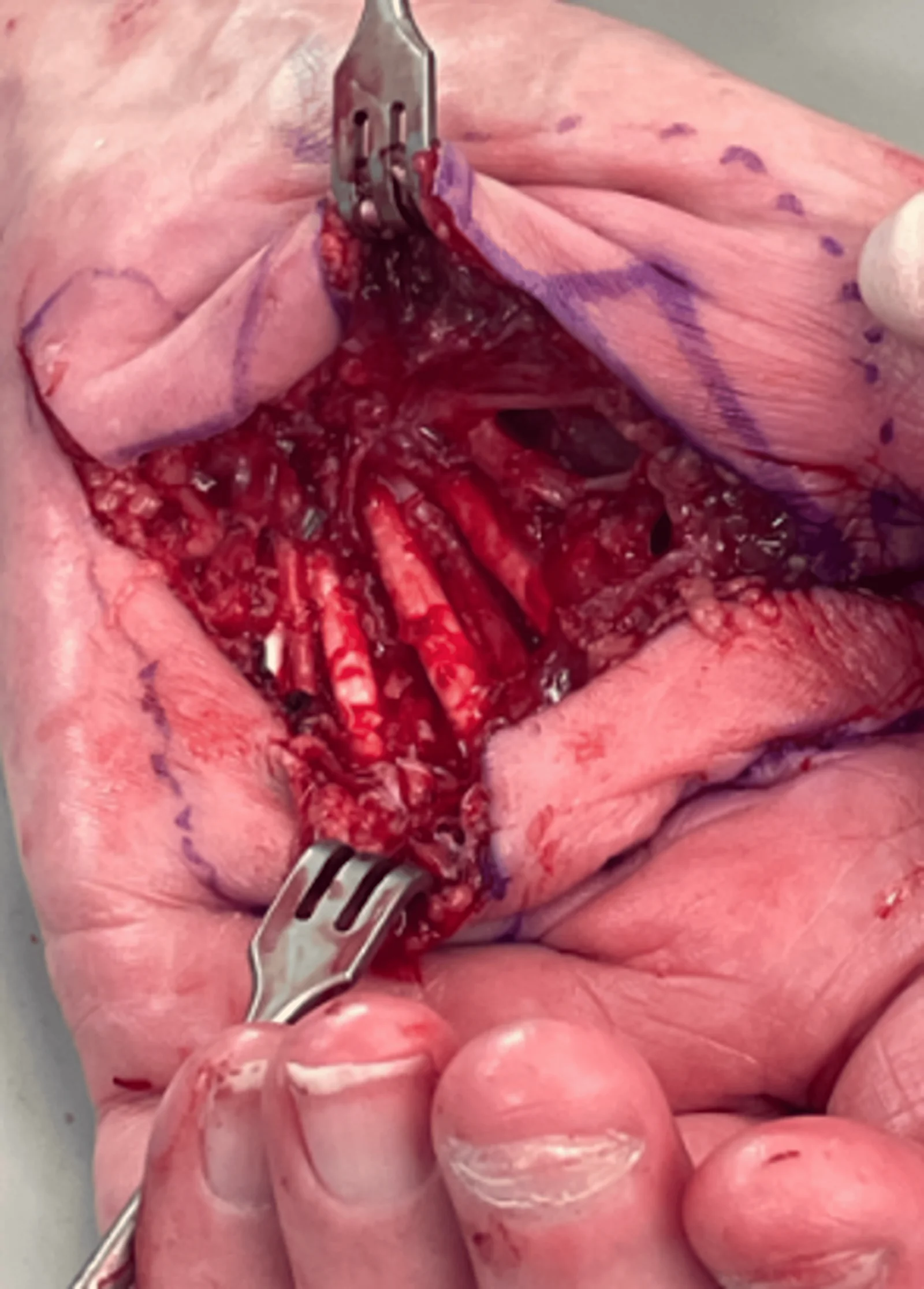
Intraoperative view following tumor excision demonstrating preservation of the common digital nerves and neurovascular bundles.

Results

Histopathological examination confirmed an ALT, a well-defined, lobulated adipocytic neoplasm measuring 80 × 63 × 25 mm and weighing 51.46 g. Microscopically, the lesion was composed predominantly of mature adipocytes exhibiting variation in cell size, with fibrous septa containing occasional atypical hyperchromatic stromal cells. No tumor necrosis, mitotic figures, or dedifferentiation was identified. The tumor was surrounded by a thin fibrous capsule, which was present at the inked margins in all sections examined (Figure 5). The excision was, therefore, reported as a marginal (shelled-out) excision. Fluorescence in situ hybridisation (FISH) analysis demonstrated MDM2 gene amplification, supporting the diagnosis of ALT.

**Figure 5:**
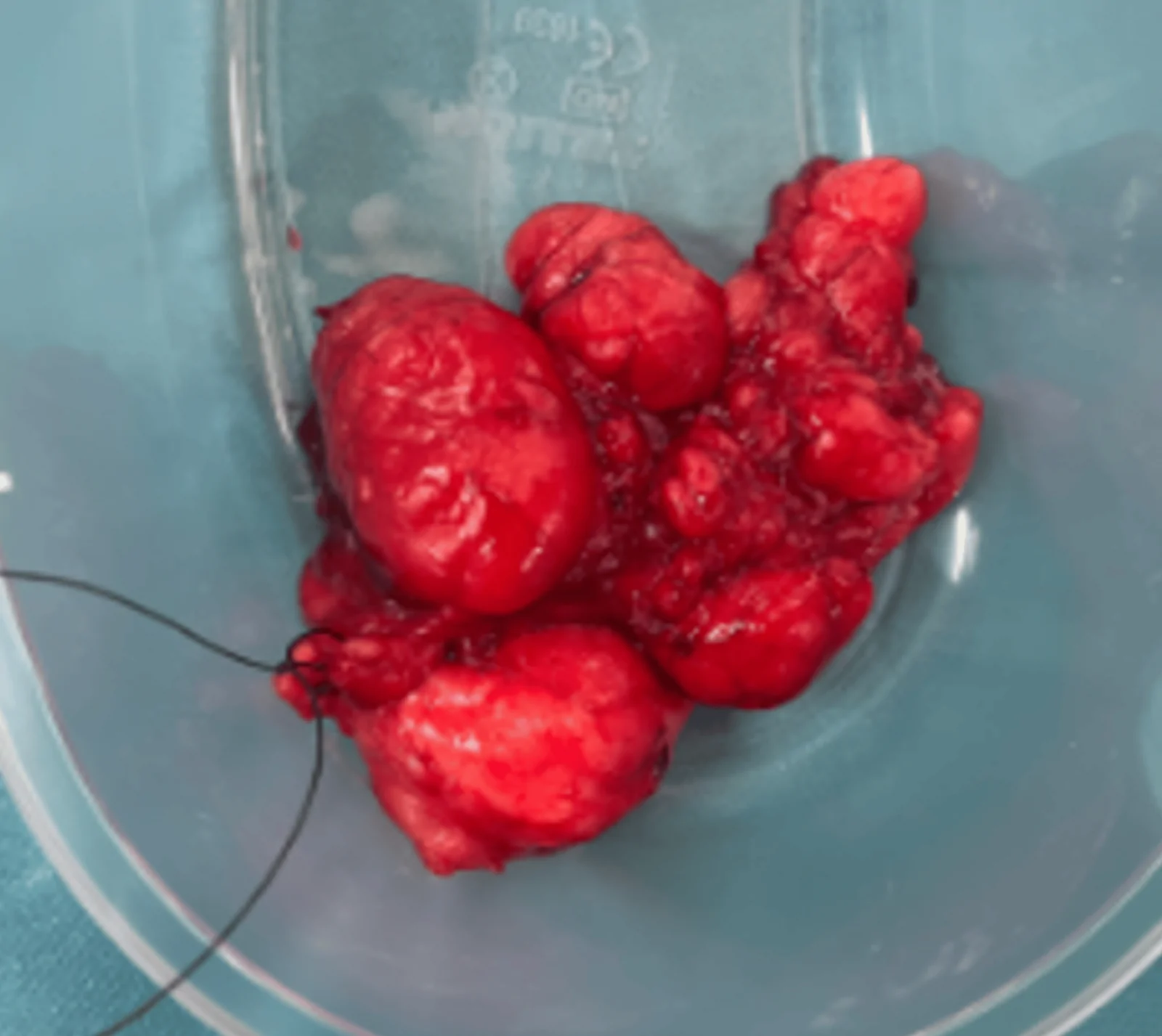
Gross specimen following complete en bloc excision of the tumor.

The patient was discharged on the day of surgery with no immediate postoperative complications. Subsequent Sarcoma MDT review recommended clinical surveillance for two years, including interval MRI at six months.

At three-month follow-up, the surgical wound was well healed with no evidence of infection or wound complications. Clinical examination demonstrated intact median and ulnar nerve motor and sensory function, with no neurovascular deficits identified. Hand function was preserved, and there was no clinical evidence of local recurrence at three months postoperatively. Following review by the Sarcoma MDT, ongoing surveillance was recommended, consisting of six-monthly clinical follow-up for two years, with a six-month postoperative MRI scan scheduled as part of the surveillance plan.

## Discussion

ALTs rarely arise in the hand, and simultaneous extension into both the carpal tunnel and Guyon's canal has only rarely been reported in the literature. Owing to the confined anatomy of the palm and the close relationship of these tumors to the median and ulnar neurovascular structures, surgical excision presents significant technical challenges. In our patient, despite the tumor's considerable size and extension into both anatomical compartments, there were no preoperative neurological deficits, highlighting the indolent nature of ALT and its potential to remain clinically silent until substantial enlargement. This emphasises the importance of maintaining a broad differential diagnosis when evaluating slowly enlarging palmar masses, particularly when clinical findings appear disproportionate to the lesion size [4].

MRI is the imaging modality of choice for evaluating lipomatous tumors, providing accurate assessment of tumor size, anatomical extent, and its relationship to surrounding neurovascular structures. Features suggestive of ALT include large size, deep location, thick internal septa, and areas of signal heterogeneity. However, imaging alone cannot reliably distinguish ALT from benign lipoma, making histopathological examination with MDM2 amplification by FISH essential for definitive diagnosis [5]. Preoperative MRI was particularly valuable in this case by demonstrating extension into both the carpal tunnel and Guyon's canal, thereby facilitating operative planning and anticipation of the close relationship between the tumor and the surrounding neurovascular structures [6].

The primary treatment for ALT is complete surgical excision while preserving function whenever possible. In anatomically constrained regions such as the hand, wide excision may compromise critical neurovascular structures without improving outcomes. Mussi et al. demonstrated that marginal excision provides satisfactory local tumor control while avoiding unnecessary sacrifice of major nerves and vessels. In the present case, meticulous microsurgical dissection allowed complete en bloc marginal excision with preservation of the median and ulnar neurovascular structures and excellent postoperative hand function [7]. This case also highlights the importance of careful intraoperative identification and protection of the digital nerves and vascular bundles, as preservation of these structures is essential for maintaining postoperative hand function and minimising morbidity.

Although ALT has no metastatic potential unless dedifferentiation occurs, local recurrence remains the principal concern, particularly following marginal excision. Consequently, ongoing clinical and radiological surveillance is recommended. Long-term follow-up is especially important because local recurrence may occur several years after the initial excision, even in the absence of aggressive histological features. Early recognition of recurrence allows timely intervention while preserving hand function and limiting the need for more extensive surgery [8]. In this case, the Sarcoma MDT recommended six-monthly clinical follow-up for two years, with interval MRI at six months as part of the planned surveillance. This two-year surveillance period reflects the current MDT follow-up plan and does not imply that the risk of late local recurrence is limited to this period; longer-term follow-up may be guided by local sarcoma surveillance protocols and the patient’s clinical course.

This case demonstrates that even large palmar ALTs extending into both the carpal tunnel and Guyon's canal can be successfully managed with careful preoperative planning, the Sarcoma MDT input, and nerve-sparing surgical excision while maintaining excellent functional outcomes. Given the rarity of this anatomical presentation, this report adds to the limited literature describing the surgical management of giant palmar ALTs involving both major fibro-osseous tunnels of the wrist and highlights the importance of balancing oncological clearance with functional preservation.

## Conclusions

This case demonstrates that even large palmar ALTs extending simultaneously into the carpal tunnel and Guyon's canal can be successfully managed with meticulous nerve-sparing marginal excision. Comprehensive preoperative imaging, multidisciplinary sarcoma assessment, and careful microsurgical dissection are essential to achieve local tumour control while preserving hand function.

ALT should be included in the differential diagnosis of large, slowly enlarging volar hand masses, particularly when MRI demonstrates atypical lipomatous features. MRI is essential for defining tumour extent and operative planning when neurovascular structures may be involved. Sarcoma MDT review facilitates appropriate oncological management. Nerve-preserving en bloc excision can achieve tumor clearance while maintaining hand function, even in anatomically challenging locations.
